# One new species and two new records of the genus *Aeolothrips* from Iran (Insecta, Thysanoptera, Aeolothripidae)

**DOI:** 10.3897/zookeys.557.7046

**Published:** 2016-01-28

**Authors:** Jalil Alavi, Mehdi Modarres Awal, Lida Fekrat, Kambiz Minaei, Shahab Manzari

**Affiliations:** 1 Department of Plant Protection, College of Agriculture, Ferdowsi University of Mashhad, Iran; 2 Department of Plant Protection, College of Agriculture, Shiraz University, Iran; 3 Insect Taxonomy Research Department, Iranian Research Institute of Plant Protection, Agricultural Research, Education and Extension Organization (AREEO), Tehran, Iran

**Keywords:** Aeolothrips, Iran, new record, new species

## Abstract

*Aeolothrips gundeliae* **sp. n.** is described, and two bicolored species of the same genus, *Aeolothrips ericae* Bagnall and *Aeolothrips albithorax* Pelikan are newly reported from northeast of Iran. Diagnostic characters are provided for each species as well as illustrations to distinguish these species.

## Introduction

Most species in the order Thysanoptera are placed in one of the two families, Phlaeothripidae or Thripidae. Aeolothripidae, with more than 202 extant species and 23 genera, is ranked as the second largest family of suborder Terebrantia after Thripidae (ThripsWiki 2015). Aeolothripids are mainly distributed in the temperate parts of the world, although members of several genera are restricted to the tropics. Those are mainly flower living phytophagous specices, or facultative predators of other arthropods (Reynaud 2010). A few species can be found living at ground level as obligate predators (ThripsWiki 2015). Approximately 60% of the described species in this family are placed either in the Holarctic genus *Aeolothrips* Haliday or in the Australian genus *Desmothrips* Hood, with 103 and 20 species respectively (ThripsWiki 2015). The remaining known species of this family are distributed between 21 genera.

In Iran, the main aeolothripid genus, *Aeolothrips*, comprises many species (Minaei 2013a). There has recently been a remarkable increase in the number of taxonomic studies on this genus, with the number of species known from Iran increasing from 12 (Bhatti et al. 2009) to 17 (Minaei 2013b), and with four new species in the most recent studies (Minaei 2014, 2015; Alavi et al. 2015). In this paper one further new species of *Aeolothrips* is described from Iran.

## Material and methods

The specimens were collected from various places of the northeastern province of Iran, Khorasan-e shomali, during spring of 2014, by shaking or beating flowers onto a white plastic tray. The fallen thrips were then removed from tray surface into the vials containing 95% alcohol using a fine brush. Thrips specimens were mounted onto slides in Canada balsam by minor changes in protocol given by Bisevac (1997). Morphological terminology follows that of Mound and Marullo (1998) and zur Strassen (2003). All measurements were made with a Micros MCX100 microscope; measurements in descriptions are given in micrometers. Photomicrographs were captured using a Motic BA310 microscope with Motic Image Plus 2.0ML software.

**Type deposition.** The female holotype and one male paratype of *Aeolothrips gundeliae* sp. n., one female and one male of *Aeolothrips albithorax*, and two females of *Aeolothrips ericae* are deposited in Hayk Mirzayans Insect Museum (HMIM), Iranian Research Institute of Plant Protection (IRIPP), Tehran. Furthermore, one paratype female and one paratype male of the new species are deposited in the Senckenberg Natural History Museum, Frankfurt.

## Taxonomy

### Aeolothrips albithorax

Taxon classificationAnimaliaThysanopteraAeolothripidae

Pelikan, 1964

Figs 1–8

#### Note.

Described from Tajikistan (central Asia), this is the first report of this species outside its type locality. Collected originally from “low herbages” and “*Rumex* sp.” (Pelikan 1964), we collected it only on *Crambe cordifolia*.

#### Material examined.

IRAN, Khorasan-e shomali province, from flowers of *Crambe cordifolia* (Brassicaceae), all collected by J. Alavi: 11 females, 3 males, Bojnourd, Ghuch-ghaleh village, 16 April 2014; 1 male, Bojnourd, Rakhtian village, 21 April 2014; 1 female, Esfarayen, Pelmis spring, 27 April 2014; 1 female, Bojnourd, Chahar-kharvar village, 4 May 2014.

#### Diagnosis.

**Figures 1–8.**
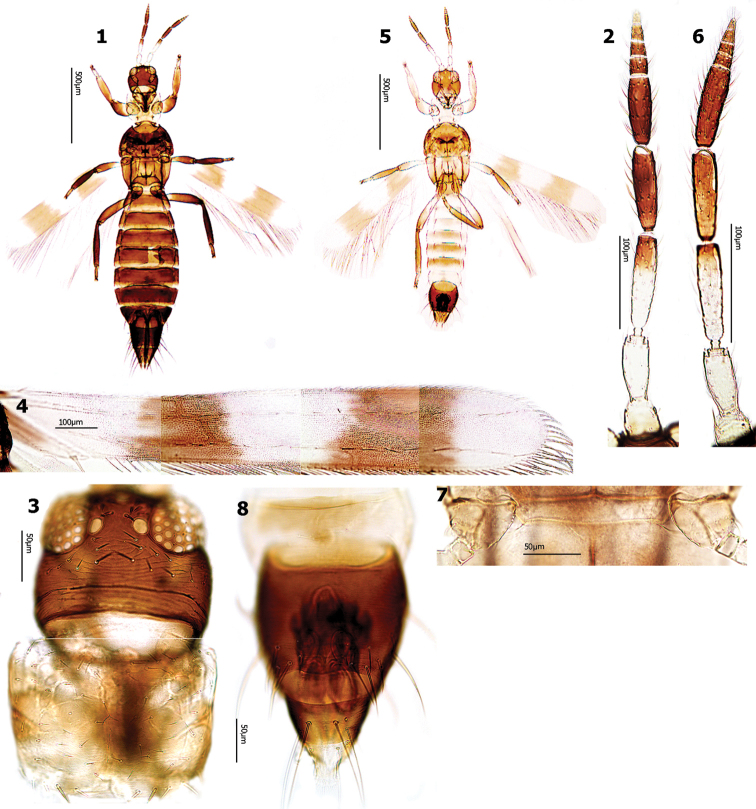
*Aeolothrips albithorax.* Female: (**1–4**): **1** Body **2** Antenn **3** Head & pronotum **4** Fore wing. Male (**5–8**): **5** Body **6** Antenna **7** Middle coxae **8** Abdominal tergites VIII–X.

Female distinctly bicolored, lemon yellow prothorax in sharp contrast to the rest of the dark brown body (Fig. 1); legs brown. Antennal segment I yellowish grey, II and III yellow, III rather abruptly brown in distal half (Fig. 2). Submedian pair of posteromarginal setae on pronotum longer and stouter than others (Fig. 3). Fore wings with two brown cross bands, connected with dark posteromarginal vein between them (Fig. 4).

Males generally similar to females but paler and smaller (Figs 5–6). Middle coxae without stridulatory structure (Fig. 7). Abdominal tergites IV–VI without dorsal tubercles. Segment IX without claspers, posterior margin of tergite IX convex medially (Fig. 8).

### Aeolothrips ericae

Taxon classificationAnimaliaThysanopteraAeolothripidae

Bagnall, 1920

Figs 9–18

#### Note.

Described from England on flowers of *Erica tetralix*, this species is widespread across western Eurasia, and introduced to North America (zur Strassen 2003, Hoddle et al. 2015). It is usually found on flowering Ericaceae (*Erica* and *Calluna*) but also on various Fabaceae (zur Strassen 2003, Hoddle et al. 2015). This is the first record of this species from Iran.

#### Material examined.

IRAN, Khorasan-e shomali province, all collected by J. Alavi: 1 female, Bojnourd, Oter-abad village, from flowering *Paliurus spina-christi* (Rhamnaceae), 12 May 2014; 1 female, Ashkhaneh, Biyar falls, from flowering *Glycyrrhiza glabra* (Fabaceae), 30 May 2014; 1 female, same location and date, from flowering *Conium maculatum* (Apiaceae). 4 females, Ashkhaneh, Darkesh village, from flowering *Rorippa officinale* (Brassicaceae), 30 May 2014; 2 females, same location and date, from flowering *Paliurus spina-christi*; 1 female, Ashkhaneh, Hawer village, from flowering *Cornus sanguinea* (Cornaceae), 30 May 20; 3 females, same location and date, from flowering *Melilotus officinalis* (Fabaceae). GERMANY, 1 female, Baden-Württemberg, Reichenbach, from herbs and grasses, 1 June 2012, collected by M. Ulitzaka. NORWAY, all collected by S. Kobro: 1 female and 1 male, Haoya, from *Lathyrus pratensis* (Fabaceae), 29 June 1996; 1 female, Aurland, from *Galium verum* (Rubiaceae), 30 June 1998; 1 female, Aurland, from *Lathyrus pratensis*, 30 June 1998; 1 female, Fagerstrand, from *Lathyrus pratensis*, 5 July 1998; 1 male, Eidfjord, from *Lotus corniculatus* (Fabaceae), 31 May 1999; 1 female, Steigen, from *Vicia cracca* (Fabaceae), 14 July 1999; 1 male, Horten, from *Vicia cracca*, 2 July 1999.

#### Diagnosis.

**Figures 9–18.**
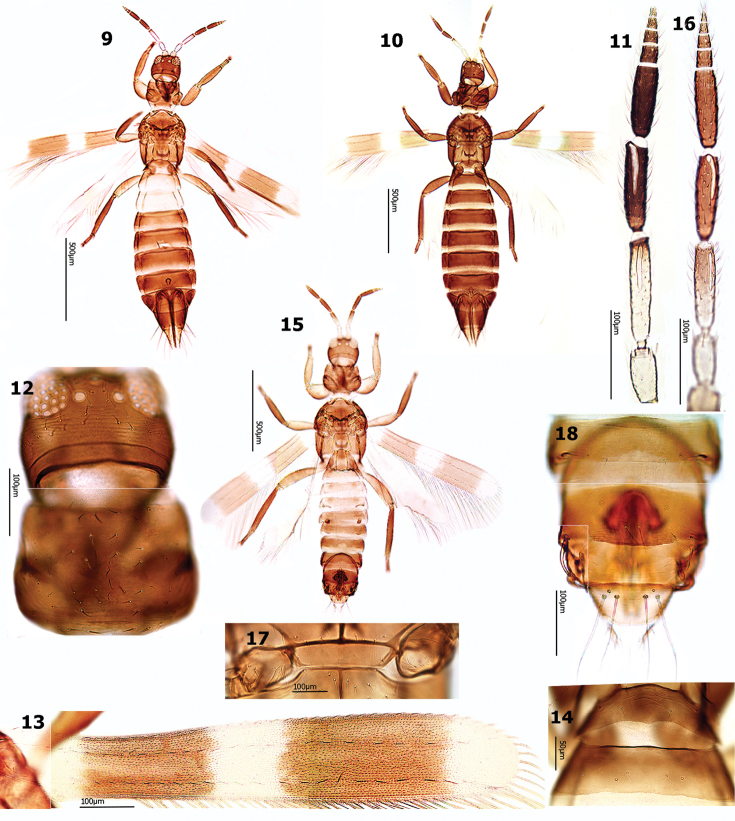
*Aeolothrips ericae*. Female (**9–14**): **9–10** Body **11** Antennal segments II–XI **12** Head & pronotum **13** Wing **14** Abdominal tergites I–II. Male (**15–17**): **15** Body **16** Antenna **17** Middle coxae **18** Abdominal tergites VIII–X.

Female distinctly bicolored, generally brown with abdominal segment II and/or III yellow to yellowish brown (Fig. 9), sometimes (in European specimens) all abdominal segments uniformly brown (Fig. 10), segment X orange-yellow, much paler than VIII–IX (Figs 9–10); Antennal segment I greyish yellow, II–III yellow, III brown in distal one third (Fig. 11). Pronotum with about 40 discal setae (Fig. 12). Fore wings with two separate long brown cross bands, 2–3 times as long as intervening white area (Fig. 13). Abdominal tergite I without campaniform sensilla (Fig. 14).

Males paler and smaller than females (Fig. 15–16). Middle coxae with stridulatory structure (Fig. 17). Abdominal tergites III–VIII with dorsal tubercles. Segment IX with bifurcate claspers and sickle–shaped setae laterally (Fig. 18).

#### Remarks.

The bicolored body pattern in some specimens of *Aeolothrips ericae* makes the species resemble only *Aeolothrips albicinctus* Haliday, but it is distinguished from that ant-mimic species by its well-developed wings (*versus* usually short wings) and shorter and stouter antenna. Moreover, males of *Aeolothrips ericae* with bifurcate claspers are readily distinguishable from *Aeolothrips albicinctus* males. The male of *Aeolothrips ericae* is also similar in color and structure to *Aeolothrips collaris*, but it is distinguished from the latter by having distinctly longer cross-bands on fore wings and also shorter distance of median setae S1from each other.

### Aeolothrips gundeliae sp. n.

Taxon classificationAnimaliaThysanopteraAeolothripidae

http://zoobank.org/25D34F9B-C959-4C5F-80A6-42EFA834E136

Figs 19–33

#### Material examined.

Holotype female: IRAN, Khorasan-e shomalii province, Bojnourd, Sar-cheshmeh village, from flowering *Gundelia tournefortii* (Asteraceae), 26 April 2014, collected by J. Alavi.

Paratypes: (all from IRAN, Khorasan-e shomalii province, from flowering *Gundelia tournefortii*, collected by J. Alavi): 25 females, 7 males, same data as holotype;1 female, Raz, Kargaz village, 10 May 2014; 1 female, Bojnourd, Tatar village, 12 May 2014; 2 females, Shirvan, 20 km after Lojali village, 7 June 2014.

#### Description.

*Female macroptera*. Head wider than long, cheeks convex (Fig. 22); vertex with 6–7 pairs of preocellar setae in front of ocellar triangle; postocular area with 8–9 pairs of setae in 2–3 transverse rows. Antennal segment III with straight liner sensorium, extending to apical third of segment (or more), not reaching to half length of the segment; IV with sensorium curved at apex, extending at most to basal half of the segments, surpassing extreme distal tip of segment (Fig. 20).

**Figures 19–33.**
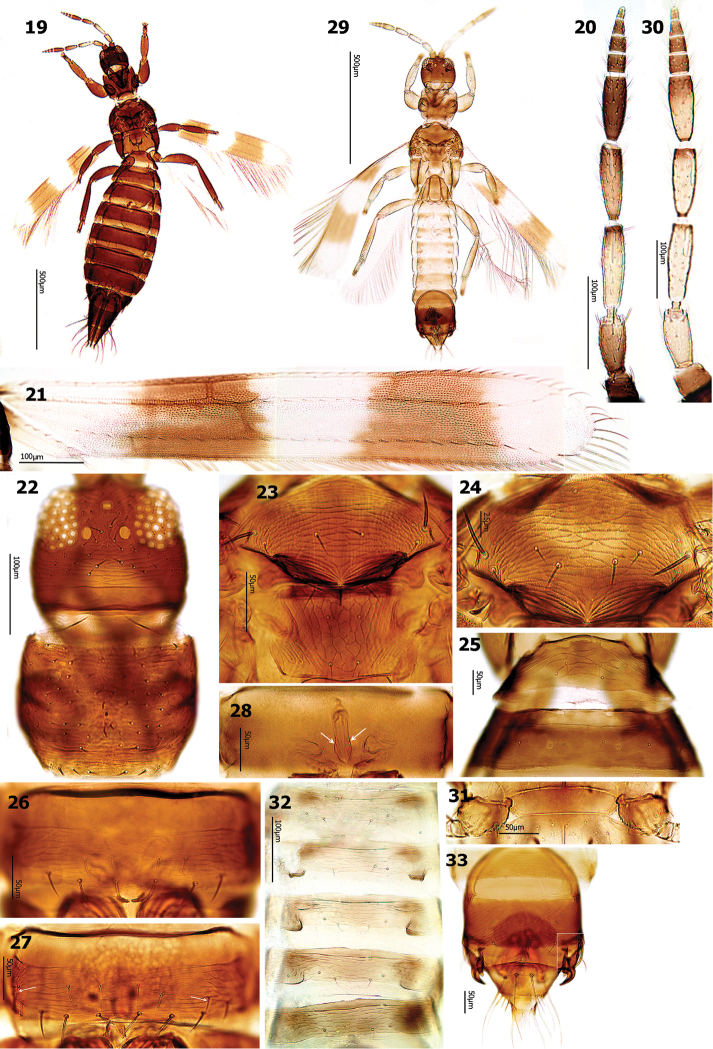
*Aeolothrips gundeliae* sp. n. Female (**19–33**): **19** Body **20** Antenna **21** Fore wing **22** Head & pronotum **23** Meso- and metanotum (Holotype) **24** Mesonotum (Paratype) **25** Abdominal tergites I–II **26** Abdominal sternite VII (Holotype) **27** Abdominal sternite VII (Paratype, arrows indicate lateral discal seta) **28** Spermatheca (arrows indicate spiniform chitinous processes). Male (**29–33**): **29** Body **30** Antenna **31** Middle coxae **32** Abdominal tergites III–VI **33** Abdominal tergites VIII– X.

Pronotum distinctly sculptured, with about 50 small setae, with 5–6 pairs of posteromarginal setae (Fig. 22). Mesonotum with 1 pair of median setae (Fig. 23), in a few paratypes with 3–4 median setae (Fig. 24). Metanotum with equiangular reticulation medially, without internal markings (Fig. 23). Forewing first cross vein situated in the middle of the first cross band, second cross vein at the basal part of the second cross band (Fig. 21); scale with 6–10 (usually 8) veinal setae.

Abdominal tergite I with distinct transverse striations medially and laterally (Fig. 25); . Abdominal sternites with distinct transverse striations; sternite II with 3 pairs of posteromarginal setae, median pair far from posterior margin; III–VI with 4 pairs; VII with 4 pairs of which the last lateral pair is far from posterior margin, the distance of S1 setae from each other usually approximately equals to that of S1from S2 (Figs 26–27); sternites II–VI each with 0–3 median discal setae (in holotype, II–V each with 1seta, and VI with 2 setae); sternite VII with 2 pairs of accessory setae, arranged besides each other, far from posterior margin (Fig. 27–28). In two paratypes sternite VII with 1 or 2 (one seta in each side) discal setae laterally in addition to 2 pairs of accessory setae submedially (Fig. 27). Spermatheca structurally very similar to that of *tenuicornis* (see: Bhatti 1988), but slightly smaller and thinner, with fewer number of spiniform chitinous processes (Fig. 28).

*Measurements* (holotype female in microns). Body distended length 1900. Head length (width across cheeks) 135 (171). Antenna length 420; segments I–IX length (width): 32 (22), 54 (27), 88 (24), 76 (25), 66 (25), 20 (20), 17 (17), 16 (12), 15 (7). Pronotal median length (width) 140 (220), Pterothorax ventral length (width) 350 (300). Mesonotum median setae length (interval) 17 (42), strong lateral setae length 37. Metanotum anteromarginal setae length (interval) 25 (44), posterior setae length (interval) 15 (25). Fore wings length 940, width across 1st anterior cross vein 122, across second cross vein 135, the cross bands length along the anterior margin 270 and 230–250, the intervening white area length 150. Tibia length: 165, 150, and 250. Tergite IX median length 105, S1 length 159, S2 length 171. Ovipositor length 390.

*Male macroptera*. Body pale brown (Fig. 29), sometimes seems bicolor; head prothorax and mesothorax brown, metathorax pale brown, abdominal segment I pale brown, II–VI pale brown to yellowish brown, VII–X brown. Legs yellowish brown, fore tibiae yellow, all tarsi yellow. Antennal segments I pale brown; II–IV yellow; III–IV with apical margins light brown; V–IX light brown; V lighter in distal two thirds (Fig. 30). Mesonotum with 1–3 pairs of median setae. Middle coxae with stridulatory structure (Fig. 31). Abdominal tergites IV–VI with dorsal tubercles (Fig. 32). Sternites III with 0–6; IV with 3–6; V with 3–6; VI with 2–7 and VII with 2–5 discal setae. Segment IX with bifurcate claspers, and with sickle-shaped setae laterally (Fig. 33), with dark dorsal plate rounded anteriorly, campaniform sensilla situated out of dorsal dark plate, posterior margin concave medially, semilateral setae short, only slightly surpassing the dorsal furcate claspers, two median setae S1 rather long and curved (Fig. 33).

*Measurements* (paratype male, in microns). Body distended length 1350. Head length (width across cheeks) 118 (157). Antenna length 360, segments I–IX length (width): 27 (28), 51 (20), 71–76 (20), 60 (22), 56 (23), 13 (18), 12 (15), 12 (12), 10 (6). Mesonotum median setae length (interval) 17 (26–36), strong lateral setae length 27. Fore wings length 780–840, width across 1st anterior cross vein 100, across second cross vein 115, the cross bands length along the anterior margin 120 and 160, the interval white area length 140. Abdominal tergite I length 120–127. Tergite IX median length 76, semilateral setae length (interval) 41–46 (137), length of dorsal setae S1 49, S2 25.

#### Etymology.

This species is named after the genus of plant from which it was collected.

#### Remarks.

Possession of discal setae on sternites is not usual in the genus *Aeolothrips*. This condition can be seen at least in two other aberrant species, the Indian species, *Aeolothrips moundi* Kulshrestha & Vijay Veer, which has one pair of discal setae laterally on sternite VII in female (Kulshrestha and Vijay Veer 1984), and the African species *Aeolothrips scabiosatibia* Moulton, with 2–3 pairs of discal setae laterally on sternites VI–VII in female.

Female of *Aeolothrips gundeliae* sp. n. is distinguished from *Aeolothrips moundi* by presence of discal setae on sternites II–VI (0–3) and in the same time there is no discal seta on sternite VII (except two paratypes as explained above). Moreover, they are different in mesonotal median setae (1–2 pairs *versus* 1 pair) and color of fore wing apex (white *versus* shaded). Female of *Aeolothrips scabiosatibia* especially characterized by the spiny fore tibia on dorsal side, and long pronotal posteromarginal seta. Male of the new species is distinguished from *Aeolothrips moundi* and *Aeolothrips scabiosatibia* by having claspers and having several discal setae on sternites.

The new species shares some characters with the Australian genus *Desmothrips* Hood, such as presence of discal setae on sternites as well as presence of more than one pair of mesonotal setae in some specimens. But in *Aeolothrips gundeliae* sp. n., sternal discal setae III–VI are placed medially (*versus* laterally in *Desmothrips*). Additionally, sternite VII has 2 pairs of accessory setae submedially between marginal setae S1 and S2, whereas in *Desmothrips* in addition to the marginal setae, sternite VII has discal setae laterally and sometimes medially, as well as 2 pairs of accessory setae submarginally between marginal setae S1 and S2 (Mound and Marullo 1998, Mound 1972). Finally, apex of fore wing of the new species is not shaded in contrast to *Desmothrips* species (except *Desmothrips marilynae* Mound & Marullo, 1998).

*Aeolothrips gundeliae* sp. n. was collected only on *Gundelia tournefortii* from various areas of the province. Furthermore, this species was observed in 6 of 10 samplings on this plant; so, it seems likely to be a monophagous species on this plant.

## Supplementary Material

XML Treatment for Aeolothrips albithorax

XML Treatment for Aeolothrips ericae

XML Treatment for Aeolothrips gundeliae

## References

[B1] Alavi J Modarres Awal M Fekrat L Minaei K (2015) The Holarctic genus *Aeolothrips* (Thysanoptera: Aeolothripidae) from Iran, with description two new species. Zootaxa 3972(1): 93–100. doi: 10.11646/zootaxa.3972.1.7 26249485 10.11646/zootaxa.3972.1.7

[B2] Bagnall RS (1920) Preliminary notes and descriptions of some European species of *Aeolothrips*. Entomologist’s monthly Magazine 56: 60–62.

[B3] Bhatti JS (1988) The spermatheca as a useful character for species differentiation in *Coleothrips* Haliday (Insecta: Terebrantia: Aeolothripidae). Zoology (Journal of Pure and Applied Zoology) 1(2): 111–116.

[B4] Bhatti JS Alavi J zur Strassen R Telmadarraiy Z (2009) Thysanoptera in Iran 1938–2007. An Overview. Thrips 7–8: 1–373.

[B5] Bisevac L (1997) A new method for mounting Thrips (Thysanoptera) on slides. Australian Journal of Entomology 36(3): 220–220. doi: 10.1111/j.1440-6055.1997.tb01457.x

[B6] Hoddle MS Mound LA Paris D (2015) Thrips of California 2012. http://keys.lucidcentral.org/keys/v3/thrips_of_california/Thrips_of_California.html [accessed 18 June 2015]

[B7] Kulshrestha SK Vijay Veer (1984) Two new species of Thysanoptera (Insecta) from India. Bulletin of Entomology 25(1): 33–37.

[B8] Minaei K (2013a) The genus *Aeolothrips* in Iran (Thysanoptera: Aeolothripidae) with one new species. Zootaxa 3630(3): 594–600. doi: 10.11646/zootaxa.3630.3.14 26131536 10.11646/zootaxa.3630.3.14

[B9] Minaei K (2013b) Thrips (Insecta, Thysanoptera) of Iran: a revised and updated checklist. Zookeys 330: 53–74. doi: 10.3897/zookeys.330.5939 24146555 10.3897/zookeys.330.5939 PMC3800805

[B10] Minaei K (2014) New record of predatory thrips, *Aeolothrips melaleucus* (Thysanoptera, Aeolothripidae) from Iran. Linzer biologische Beiträge 46(1): 637–642.

[B11] Minaei K (2015) A new species of the genus *Aeolothrips* (Thysanoptera: Aeolothripidae) from Iran. Biologia 70(10): 1401–1405. doi: 10.1515/biolog-2015-0158

[B12] Mound LA (1972) Further studies on Australian Aeolothripidae (Thysanoptera). Journal of the Australian Entomological Society 11(1): 37–54. doi: 10.1111/j.1440-6055.1972.tb01603.x

[B13] Mound LA Marullo R (1998) Biology and identification of Aeolothripidae (Thysanoptera) in Australia. Invertebrate Taxonomy 12: 929–950. doi: 10.1071/IT97014

[B14] Pelikan J (1964) Five new Thysanoptera from Soviet Central Asia. Časopis Československé Společnosti Entomologické (Acta Societatis Entomologicae Cechosloveniae) 61(3): 224–237.

[B15] Reynaud P (2010) Thrips (Thysanoptera). In: Roques A et al. (Eds) Alien terrestrial arthropods of Europe. BioRisk 4(2): 767–791. doi: 10.3897/biorisk.4.59

[B16] ThripsWiki (2015) ThripsWiki-providing information on the World’s thrips. http://thrips.info/wiki/ [accessed 18 June 2015]

[B17] zur Strassen R (2003) Die terebranten Thysanopteren Europas und des Mittelmeer-Gebietes. Die Tierwelt Deutschlands 74: 1–271.

